# Supplementary material for: Heat extremes in Western Europe increasing faster than simulated due to atmospheric circulation trends
Source: Nat Commun. 2023 Oct 26;14:6803. doi: 10.1038/s41467-023-42143-3 (PMC10603106; doi:10.1038/s41467-023-42143-3)
Supplement: Supplementary file 1 — Supplementary Information [file 41467_2023_42143_MOESM1_ESM.pdf]

Supplementary Information

Heat extremes in Western Europe increasing faster than simulated due to atmospheric circulation trends

Robert Vautard[1], Julien Cattiaux[2], Tamara Happé[3], Jitendra Singh [4], Rémy Bonnet[1], Christophe Cassou[5], Dim Coumou[3,1,6], Fabio D'Andrea[7], Davide Faranda[8], Erich Fischer[4], Aurélien Ribes[2], Sebastian Sippel [4], Pascal Yiou[8]

[1] Institut Pierre-Simon Laplace, CNRS, Université Paris-Saclay, Sorbonne Université, France
[2] Centre National de Recherches Météorologiques, Université de Toulouse, Météo-France, CNRS, Toulouse, France.
[3] Institute for Environmental Studies, Vrije Universiteit Amsterdam, Amsterdam, Netherlands
[4] Institute for Atmospheric and Climate Science, ETH Zurich, Zürich, Switzerland
[5] Centre Européen de Recherche et de Formation Avancée en Calcul Scientifique, CNRS UMR 5318, Toulouse, France
[6] Royal Netherlands Meteorological Institute (KNMI), De Bilt, Netherlands
[7] Laboratoire de Météorologie Dynamique, IPSL, CNRS, Paris, France
[8] Laboratoire des Sciences du Climat et de l'Environnement, UMR 8212 CEA-CNRS-UVSQ, Université Paris-Saclay and IPSL, 91191 Gif-sur-Yvette, France

Observation and model data

We used ERA5 reanalysis of daily maximum temperatures and streamfunction fields. Streamfunction is calculated from u- and v- wind fields at 500 hPa on a T127 Gaussian grid, and then interpolated on a 1x1 regular grid, following:
$u = -\frac{\partial \psi}{\partial y}$, and $v = \frac{\partial \psi}{\partial x}$, where ψ is the streamfunction, u is the zonal- and v the meridional component of the wind fields.

Surface daily maximum temperatures from ERA5 are interpolated to a 0.5x0.5 grid. We also used observations from the E-OBS dataset v24e [20] for daily maximum temperature (TX). E-OBS was initially taken from a 0.25 x 0.25 grid and projected onto the 0.5x0.5 grid. When considering averages over the selected Western Europe area [5W-15E;45N-60N], data are masked using the E-OBS land/sea mask (see below).

Daily maximum temperatures and streamfunction are also calculated from model simulations including all first members of each CMIP6 model ensemble. In order to increase as much as possible the estimation of capacity of models to simulate TXx and TXm trends, we used all possible CMIP6 simulations made available through the ESGF infrastructure. When considering only TXx and TXm calculations for Figure 3, we used 273 simulations made with 36 different models (see Figure 3). For Figure 4 and the analogue analysis for models, we keep only 32 models and 1 realization for which we have simultaneous 500 hPa wind fields and daily maximum temperatures.

To have an historical time series to be compared with reanalysis or observations, we concatenate historical and SSP5-8.5 scenarios available (from 2015 to 2022). Initial tests made with SSP2-4.5 showed that results presented here are insensitive to this choice.

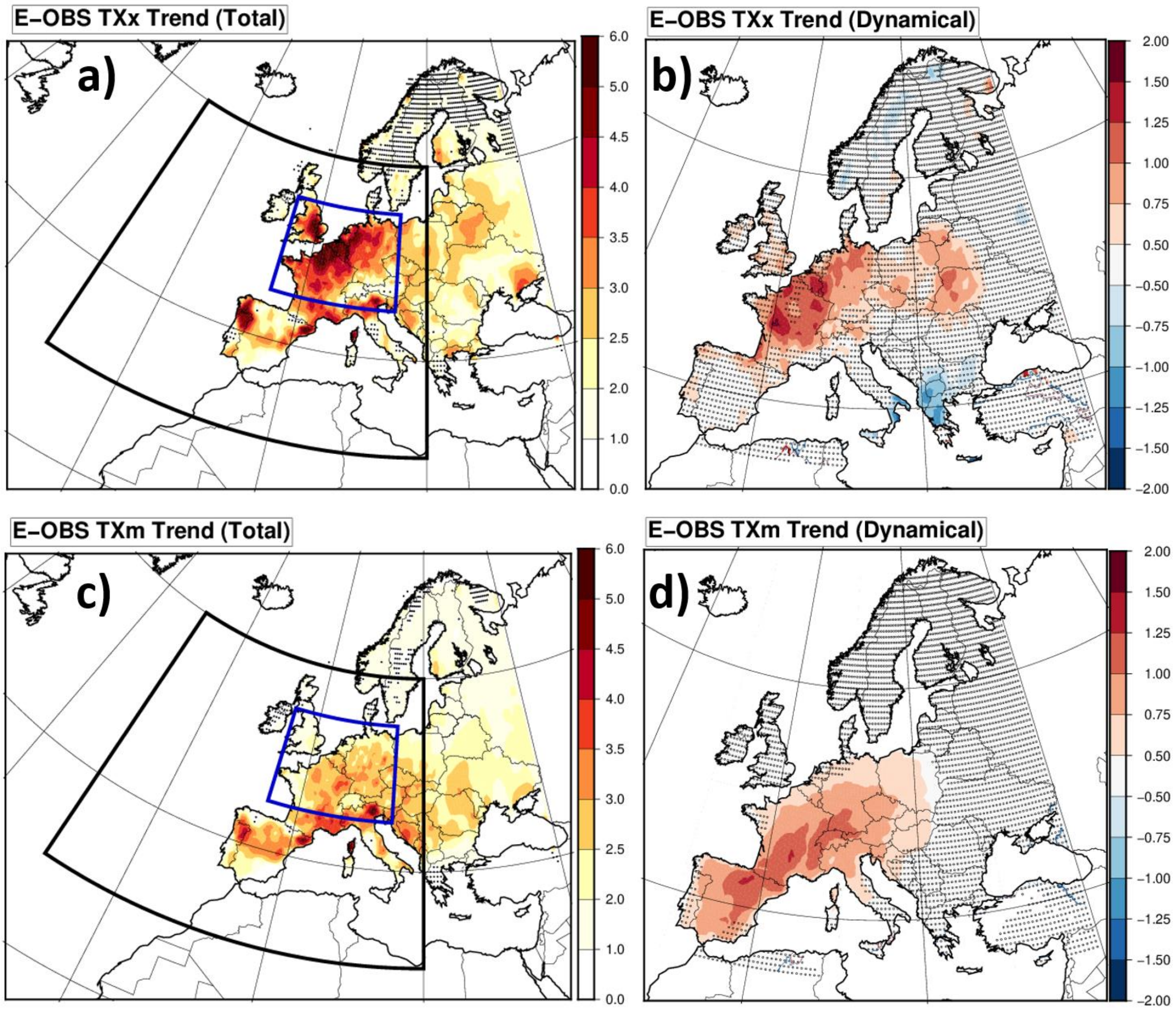

Supplementary Figure 1: **TXx trends from the E-OBS observations**

Same as Figure 1 but for E-OBS maximum daily temperatures

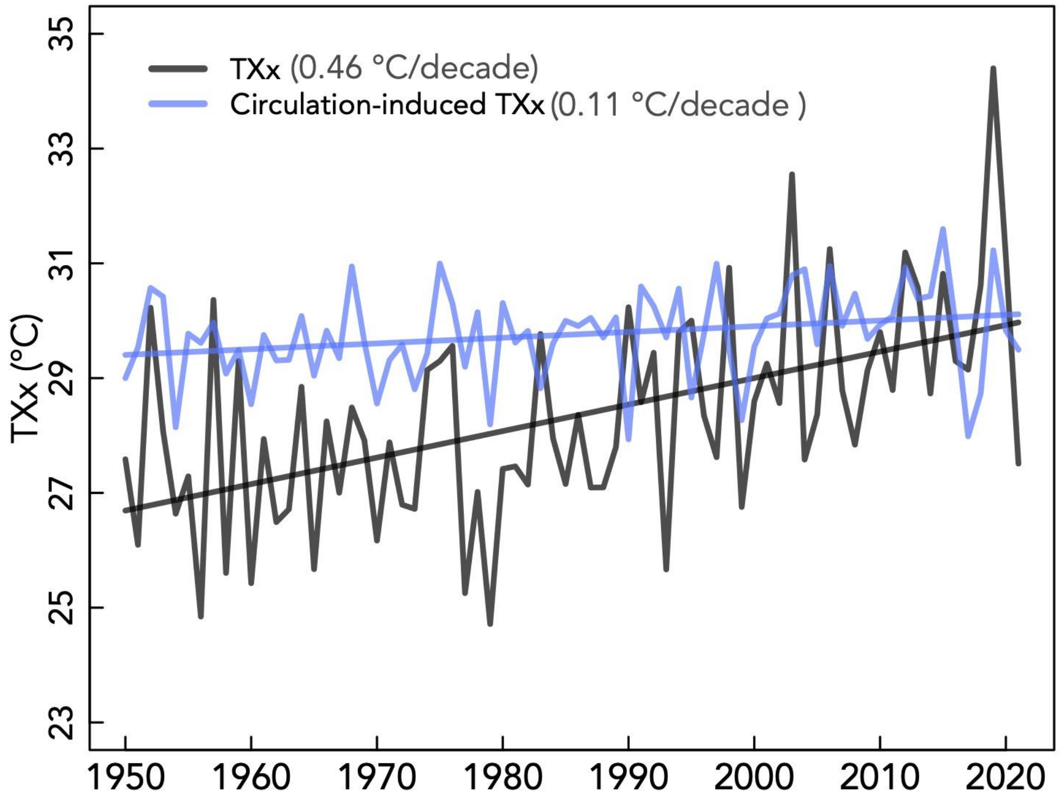

Supplementary Figure 2: **Dynamical contribution to forced TXx trend in Western Europe** Black and blue lines present the area-averaged Summer maximum temperature TXx and circulation-induced TXx over western Europe (5-15E, 45-55N), respectively. The values in parenthesis indicate the trend in the corresponding TXx time series. The trends are estimated based on Sen's slope estimator.

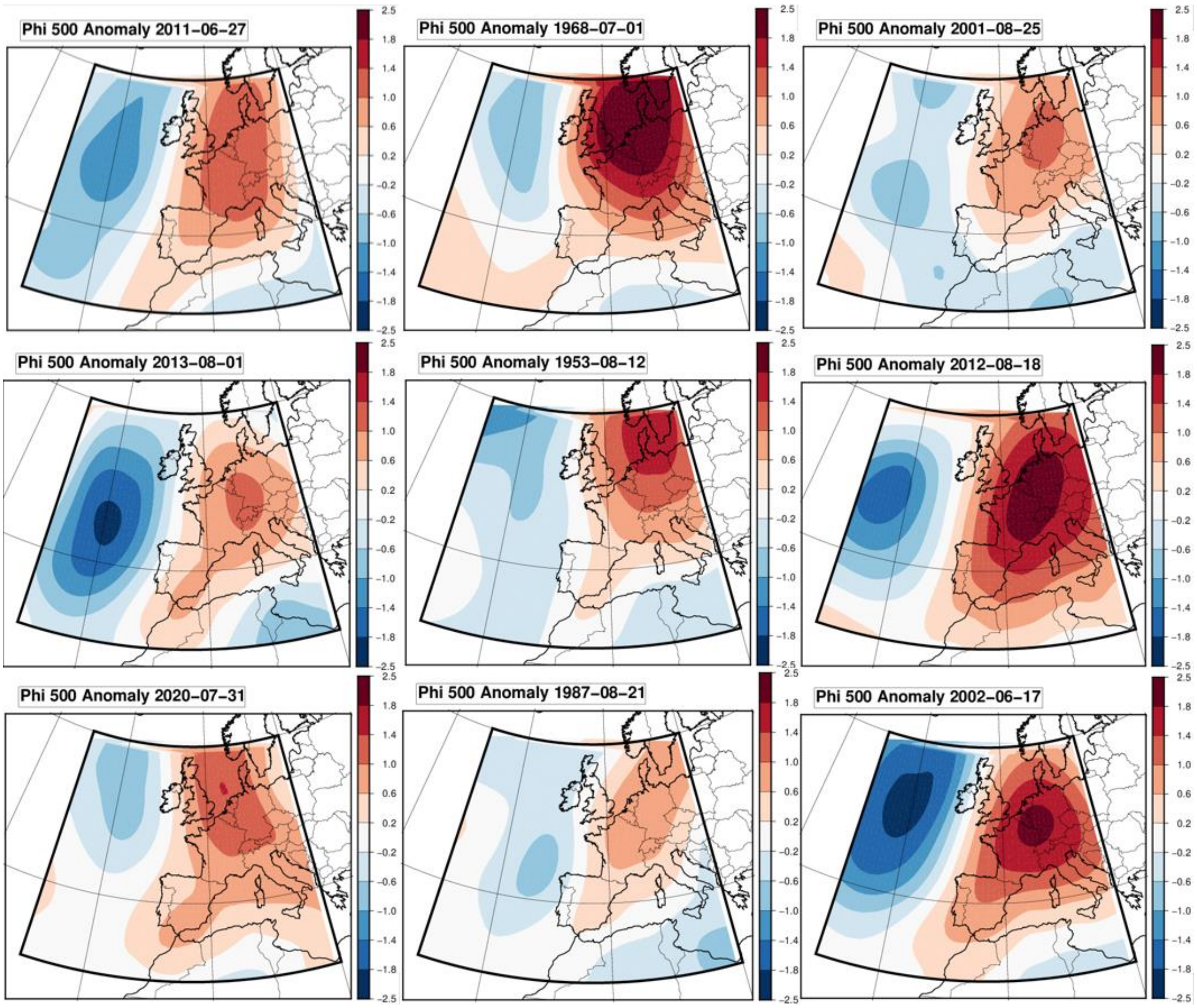

Supplementary Figure 3: Most representative hot anomaly patterns

500 hPa streamfunction anomalies (Phi 500) of the 9 most representative circulations, beyond 29/06/2019, when TXx is reached over Central France [1.5E-46.5N], by decreasing order of representativeness.

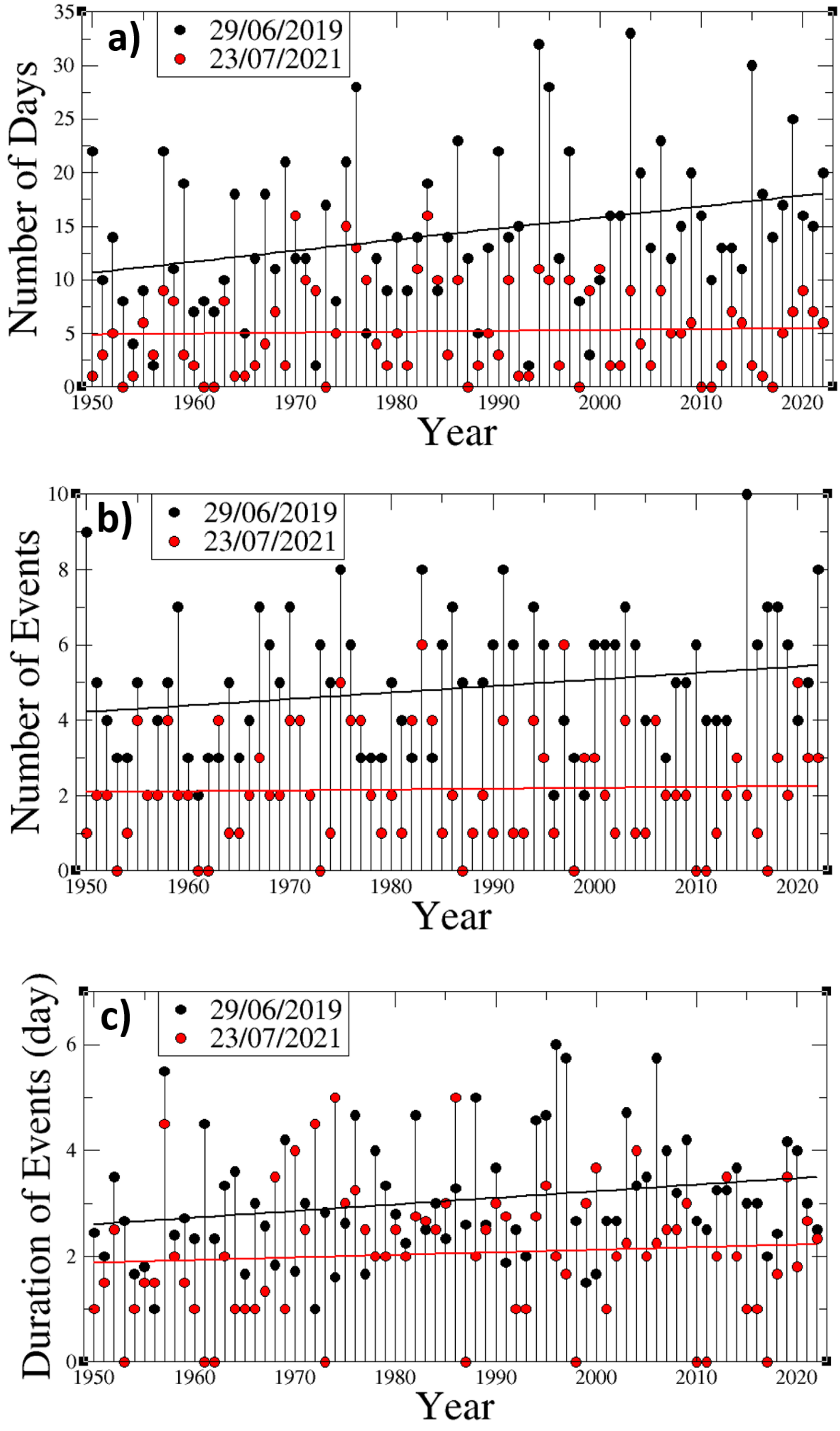

Supplementary Figure 4: Evolution of Southerly flow patterns

Evolution of the yearly number of days (a), number of events (b) and mean duration of events (c) (0 when no event found) for Southerly Flow patterns (black) (streamfunction anomalies with an ACC with the 29/06/2019 anomaly greater than 0.5). For comparison, the figure also shows (in red) the same statistics but for another pattern, that of the anomaly of the 23/07/2021, which corresponds to the date of TXx for 2021 in central France.

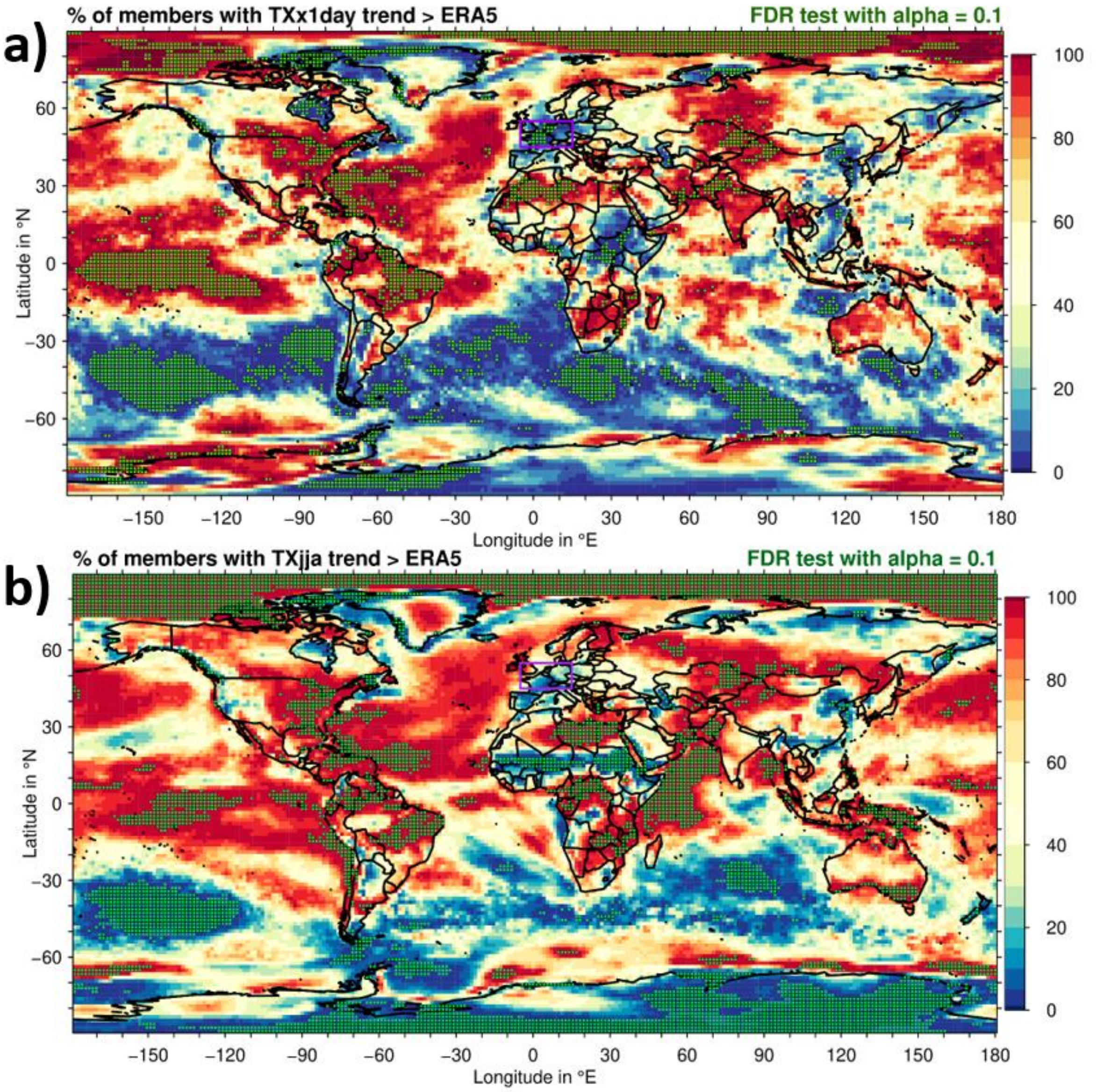

Supplementary Figure 5: **Generalization of Figure 3 at global scale**

Percentage of simulations with a trend larger than ERA5 at each grid point for (a) the annual maximum of TX (TXx) and (b) the JJA mean of TX (TXm). Green stippling indicates grid points where the mismatch between observed and simulated trends is significant at the 95% confidence level in the sense of the False Discovery Rate procedure (i.e. a two-sided multiple

test with alpha=0.1). The Western Europe box is highlighted in magenta. For the top panel, the annual (rather than JJA in Figure 3) maximum of TX is used here to capture summer heat extremes in both hemispheres; in Western Europe annual or JJA maximum are equivalent.

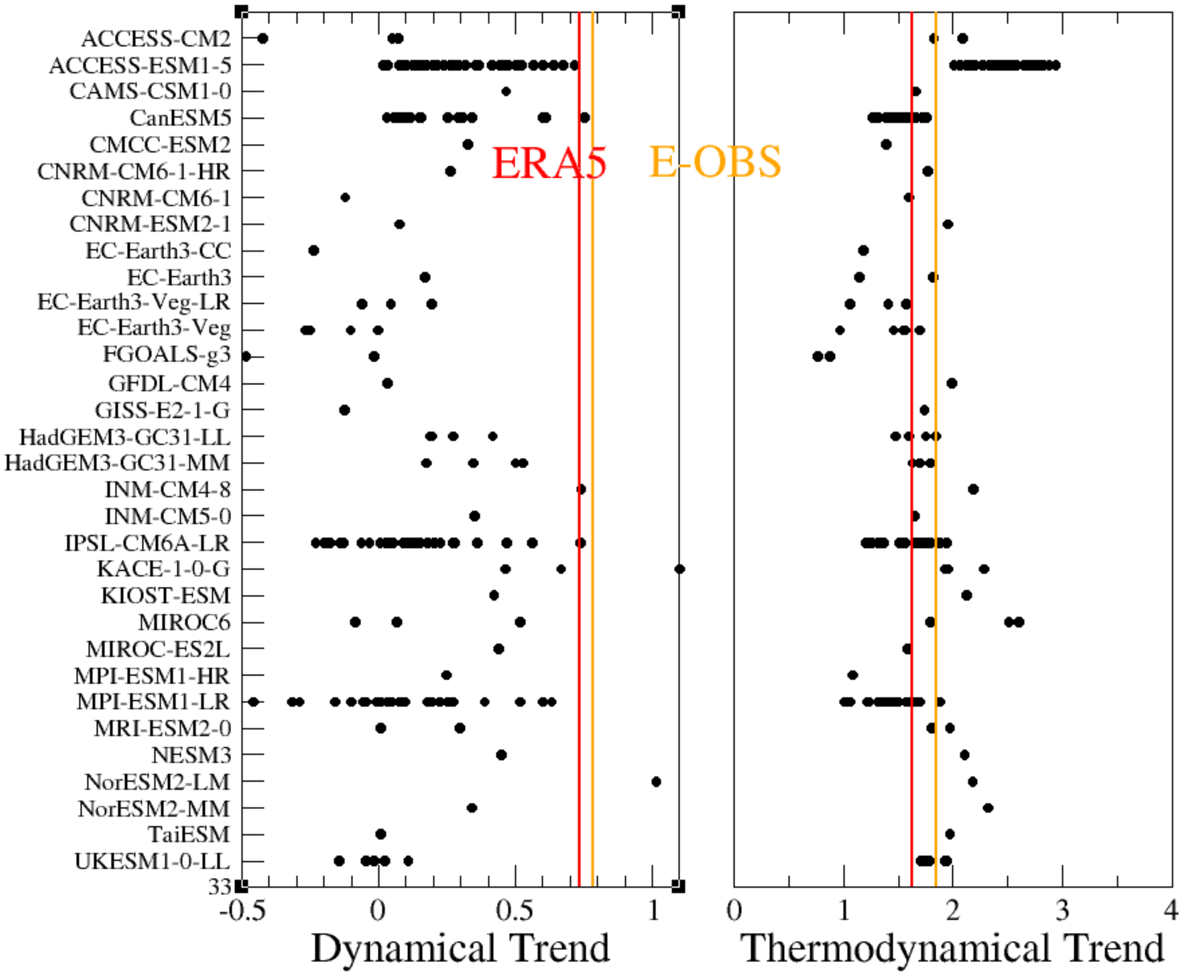

Supplementary Figure 6: **Dynamical and thermo-dynamical TXm trends**

Same as Figure 4 but for TXm instead of TXx.

Model_realization	SF frequency trends	TXx dynamical trend	Model_realization	SF frequency trends	TXx dynamic al trend
**ERA5**<br>**E-OBS**	**42.9%**	**0.79**<br>**0.86**		**42.9%**	
ACCESS-CM2_r1i1p1f1	-16.4	-0.27	HadGEM3-GC31-MM_r2i1p1f3	8.8	-0.04
ACCESS-CM2_r4i1p1f1	9.3	0.10	HadGEM3-GC31-MM_r3i1p1f3	4.8	0.59
ACCESS-CM2_r5i1p1f1	-29.4	-0.50	HadGEM3-GC31-MM_r4i1p1f3	2.4	0.46
ACCESS-ESM1-5_r10i1p1f1	25.5	0.05	INM-CM4-8_r1i1p1f1	16.4	0.26
ACCESS-ESM1-5_r1i1p1f1	11.9	0.40	INM-CM5-0_r1i1p1f1	0.7	0.24
ACCESS-ESM1-5_r2i1p1f1	7.0	0.02	IPSL-CM6A-LR_r10i1p1f1	-5.3	-0.15
ACCESS-ESM1-5_r34i1p1f1	8.9	0.35	IPSL-CM6A-LR_r11i1p1f1	-9.5	0.15
ACCESS-ESM1-5_r3i1p1f1	17.5	0.68	IPSL-CM6A-LR_r12i1p1f1	-20.4	-0.07
ACCESS-ESM1-5_r4i1p1f1	12.6	0.12	IPSL-CM6A-LR_r13i1p1f1	14.7	0.11
ACCESS-ESM1-5_r5i1p1f1	10.0	0.36	IPSL-CM6A-LR_r14i1p1f1	21.5	0.01
ACCESS-ESM1-5_r9i1p1f1	28.2	0.19	IPSL-CM6A-LR_r15i1p1f1	-8.8	0.04
ACCESS-ESM1-5_r11i1p1f1	10.8	0.31	IPSL-CM6A-LR_r16i1p1f1	9.2	0.21
ACCESS-ESM1-5_r12i1p1f1	26.7	-0.03	IPSL-CM6A-LR_r17i1p1f1	6.8	0.58
ACCESS-ESM1-5_r13i1p1f1	15.8	0.12	IPSL-CM6A-LR_r18i1p1f1	12.1	-0.23
ACCESS-ESM1-5_r14i1p1f1	25.6	0.15	IPSL-CM6A-LR_r19i1p1f1	34.8	0.37
ACCESS-ESM1-5_r15i1p1f1	18.3	0.01	IPSL-CM6A-LR_r1i1p1f1	-1.8	0.26
ACCESS-ESM1-5_r16i1p1f1	15.3	0.31	IPSL-CM6A-LR_r20i1p1f1	-15.3	-0.29
ACCESS-ESM1-5_r17i1p1f1	32.9	0.39	IPSL-CM6A-LR_r21i1p1f1	7.5	0.16
ACCESS-ESM1-5_r18i1p1f1	37.6	0.63	IPSL-CM6A-LR_r22i1p1f1	16.2	0.26
ACCESS-ESM1-5_r19i1p1f1	-2.5	0.06	IPSL-CM6A-LR_r23i1p1f1	0.9	-0.05
ACCESS-ESM1-5_r20i1p1f1	21.2	0.31	IPSL-CM6A-LR_r24i1p1f1	-2.1	0.24
ACCESS-ESM1-5_r21i1p1f1	22.9	0.63	IPSL-CM6A-LR_r25i1p1f1	-5.3	-0.10
ACCESS-ESM1-5_r22i1p1f1	-7.3	0.23	IPSL-CM6A-LR_r26i1p1f1	-3.7	0.19
ACCESS-ESM1-5_r23i1p1f1	28.7	0.30	IPSL-CM6A-LR_r27i1p1f1	-18.1	-0.21
ACCESS-ESM1-5_r24i1p1f1	9.3	0.03	IPSL-CM6A-LR_r28i1p1f1	0.7	0.08
ACCESS-ESM1-5_r25i1p1f1	9.0	0.20	IPSL-CM6A-LR_r29i1p1f1	0.5	0.04
ACCESS-ESM1-5_r26i1p1f1	3.8	0.30	IPSL-CM6A-LR_r30i1p1f1	-2.2	-0.05
ACCESS-ESM1-5_r27i1p1f1	24.7	0.38	IPSL-CM6A-LR_r31i1p1f1	15.9	0.40
ACCESS-ESM1-5_r28i1p1f1	38.8	0.42	IPSL-CM6A-LR_r32i1p1f1	-21.5	0.07
ACCESS-ESM1-5_r29i1p1f1	25.3	0.58	IPSL-CM6A-LR_r3i1p1f1	1.4	0.15
ACCESS-ESM1-5_r30i1p1f1	32.5	0.58	IPSL-CM6A-LR_r4i1p1f1	1.7	0.13
ACCESS-ESM1-5_r31i1p1f1	27.0	0.27	IPSL-CM6A-LR_r5i1p1f1	12.4	-0.29
ACCESS-ESM1-5_r32i1p1f1	6.4	0.30	IPSL-CM6A-LR_r6i1p1f1	20.9	0.48
ACCESS-ESM1-5_r33i1p1f1	22.4	0.47	IPSL-CM6A-LR_r7i1p1f1	19.8	0.62
ACCESS-ESM1-5_r35i1p1f1	31.4	0.32	IPSL-CM6A-LR_r8i1p1f1	-1.1	0.16
ACCESS-ESM1-5_r36i1p1f1	20.0	0.26	IPSL-CM6A-LR_r9i1p1f1	-11.2	-0.01
ACCESS-ESM1-5_r37i1p1f1	15.5	-0.18	KACE-1-0-G_r1i1p1f1	17.0	0.33
ACCESS-ESM1-5_r38i1p1f1	17.2	0.10	KACE-1-0-G_r2i1p1f1	23.4	0.58
ACCESS-ESM1-5_r39i1p1f1	-13.2	0.18	KACE-1-0-G_r3i1p1f1	39.5	0.44
ACCESS-ESM1-5_r40i1p1f1	27.3	0.55	KIOSK-ESM_r1i1p1f1	8.4	0.10
CAMS-CSM1-0_r2i1p1f1	-1.0	0.13	MIROC6_r1i1p1f1	20.1	0.24
CanESM5_r10i1p1f1	-4.9	-0.11	MIROC6_r2i1p1f1	-23.4	-0.31
CanESM5_r10i1p2f1	-2.0	0.17	MIROC6_r3i1p1f1	26.6	0.60
CanESM5_r1i1p1f1	10.6	0.23	MIROC-ES2L_r1i1p1f2	13.6	0.18
CanESM5_r1i1p2f1	11.7	0.01	MPI-ESM1-2-HR_r1i1p1f1	8.0	0.00
CanESM5_r2i1p1f1	9.5	0.07	MPI-ESM1-2-LR_r10i1p1f1	-0.8	-0.04
CanESM5_r2i1p2f1	-9.1	0.05	MPI-ESM1-2-LR_r1i1p1f1	2.6	0.33
CanESM5_r3i1p1f1	-7.9	-0.05	MPI-ESM1-2-LR_r2i1p1f1	23.6	0.27
CanESM5_r3i1p2f1	1.3	0.02	MPI-ESM1-2-LR_r3i1p1f1	35.6	0.20
CanESM5_r4i1p1f1	-1.8	0.03	MPI-ESM1-2-LR_r4i1p1f1	36.1	0.25
CanESM5_r4i1p2f1	2.8	0.04	MPI-ESM1-2-LR_r5i1p1f1	-23.3	-0.37
CanESM5_r5i1p1f1	4.8	0.03	MPI-ESM1-2-LR_r6i1p1f1	-6.3	0.04
CanESM5_r5i1p2f1	1.3	0.10	MPI-ESM1-2-LR_r7i1p1f1	-26.6	-0.12
CanESM5_r6i1p1f1	25.7	0.37	MPI-ESM1-2-LR_r8i1p1f1	-21.1	-0.06
CanESM5_r6i1p2f1	16.0	0.46	MPI-ESM1-2-LR_r9i1p1f1	-19.2	-0.21
CanESM5_r7i1p1f1	19.4	0.06	MPI-ESM1-2-LR_r11i1p1f1	9.2	0.32
CanESM5_r7i1p2f1	4.7	0.18	MPI-ESM1-2-LR_r12i1p1f1	1.4	-0.07
CanESM5_r8i1p1f1	-4.2	0.03	MPI-ESM1-2-LR_r13i1p1f1	-17.3	0.11
CanESM5_r8i1p2f1	-5.0	0.19	MPI-ESM1-2-LR_r14i1p1f1	1.3	-0.43
CanESM5_r9i1p1f1	25.8	0.37	MPI-ESM1-2-LR_r15i1p1f1	34.5	0.20
CanESM5_r9i1p2f1	5.9	0.49	MPI-ESM1-2-LR_r16i1p1f1	-39.3	-0.10
CMCC-ESM2_r1i1p1f1	2.6	0.10	MPI-ESM1-2-LR_r17i1p1f1	2.1	0.09
CNRM-CM6-1-HR_r1i1p1f2	23.2	-0.09	MPI-ESM1-2-LR_r18i1p1f1	-16.0	-0.01
CNRM-CM6-1_r1i1p1f2	-10.6	0.05	MPI-ESM1-2-LR_r19i1p1f1	7.9	0.47
CNRM-ESM2-1_r1i1p1f2	6.0	0.25	MPI-ESM1-2-LR_r30i1p1f1	0.4	-0.03

**Model_realization**	**SF frequency trends**	**TXx dynamical trend**	**Model_realization**	**SF frequency trends**	**TXx dynamical trend**
EC-Earth3-CC_r1i1p1f1	-14.4	-0.33	MPI-ESM1-2-LR_r21i1p1f1	0.4	0.17
EC-Earth3_r1i1p1f1	14.2	0.16	MPI-ESM1-2-LR_r22i1p1f1	21.9	-0.05
EC-Earth3_r4i1p1f1	3.4	0.24	MPI-ESM1-2-LR_r23i1p1f1	-31.7	-0.47
EC-Earth3-Veg-LR_r1i1p1f1	-9.3	0.20	MPI-ESM1-2-LR_r24i1p1f1	-19.1	0.16
EC-Earth3-Veg-LR_r2i1p1f1	9.2	0.01	MPI-ESM1-2-LR_r25i1p1f1	-4.1	-0.17
EC-Earth3-Veg-LR_r3i1p1f1	-15.3	-0.15	MPI-ESM1-2-LR_r26i1p1f1	26.2	0.50
EC-Earth3-Veg_r1i1p1f1	-5.3	-0.12	MPI-ESM1-2-LR_r27i1p1f1	29.7	0.51
EC-Earth3-Veg_r2i1p1f1	-16.9	-0.01	MPI-ESM1-2-LR_r28i1p1f1	-4.0	0.18
EC-Earth3-Veg_r3i1p1f1	-14.6	-0.33	MPI-ESM1-2-LR_r29i1p1f1	8.7	0.23
EC-Earth3-Veg_r4i1p1f1	5.0	-0.29	MRI-ESM2-0_r1i1p1f1	10.7	-0.12
EC-Earth3-Veg_r6i1p1f1	-14.4	-0.49	MRI-ESM2-0_r5i1p1f1	16.6	0.07
FGOALS-g3_r1i1p1f1	-53.1	-0.30	NESM3_r1i1p1f1	17.8	0.25
FGOALS-g3_r4i1p1f1	-10.3	-0.11	NorESM2-LM_r1i1p1f1	42.7	0.50
GFDL-CM4_r1i1p1f1	-1.7	-0.13	NorESM2-MM_r1i1p1f1	-8.7	-0.19
GISS-E2-1-G_r1i1p1f2	-16.3	-0.39	TaiESM1_r1i1p1f1	-4.2	-0.20
HadGEM3-GC31-LL_r1i1p1f3	15.9	0.30	UKESM1-0-LL_r1i1p1f2	-13.1	0.01
HadGEM3-GC31-LL_r2i1p1f3	21.3	0.24	UKESM1-0-LL_r2i1p1f2	-21.8	-0.05
HadGEM3-GC31-LL_r3i1p1f3	3.0	0.08	UKESM1-0-LL_r3i1p1f2	-3.5	-0.12
HadGEM3-GC31-LL_r4i1p1f3	5.1	0.02	UKESM1-0-LL_r4i1p1f2	-10.5	-0.01
HadGEM3-GC31-MM_r1i1p1f3	13.6	0.45	UKESM1-0-LL_r8i1p1f2	-3.6	-0.27

Supplementary Table 1: Southerly Flow (SF) frequency trend and summer TXx dynamical trends for ERA5, E-OBS, and 170 simulations for which the daily 500 hPa wind and the maximum surface temperature fields were available for both historical and SSP5-8.5 scenarios. TXx Trends are expressed in °C/GWD and frequency trends in %/GWD.
